# Targeting Cancer-Related Inflammation: Chinese Herbal Medicine Inhibits Epithelial-to-Mesenchymal Transition in Pancreatic Cancer

**DOI:** 10.1371/journal.pone.0070334

**Published:** 2013-07-29

**Authors:** Juan Zhang, Peng Wang, Huaqiang Ouyang, Jianhua Yin, Aihua Liu, Chunzheng Ma, Luming Liu

**Affiliations:** 1 Department of Integrative Oncology, Fudan University Shanghai Cancer Center, Shanghai, China; 2 Department of Oncology, Shanghai Medical College, Fudan University, Shanghai, China; 3 Department of the Second Clinical Medical College, Henan University of TCM, Zhengzhou, China; Istituto Superiore di Sanità, Italy

## Abstract

Pancreatic cancer is an almost universally fatal disease resulting from early invasion of adjacent structures and metastasis and the lack of an effective treatment modality. Our previous studies have shown that Qingyihuaji Formula (QYHJ), a seven-herb Chinese medicine formula, had significant anti-cancer effects in pancreatic cancer. Here, we examined the effects of QYHJ on pancreatic cancer cell invasion and metastasis and the potential associated mechanism(s). We found that QYHJ inhibited both tumor growth and metastasis in nude mice with human pancreatic cancer cell xenografts. Further study indicated that QYHJ inhibited epithelial-to-mesenchymal transition (EMT), which is characterized by increased E-cadherin expression and decreased vimentin, N-cadherin and Slug expression. Interleukin 6 (IL-6), a pro-inflammatory cytokine produced mainly by macrophages, could promote cancer cell EMT and invasion. In contrast, treatment with QYHJ inhibited cancer-related inflammation in tumors by decreasing infiltration of tumor-associated macrophages and IL-6 production, thus preventing cell invasion and metastasis. These results suggested that the Chinese herbal medicine QYHJ could inhibit pancreatic cancer cell invasion and metastasis in part by reversing tumor-supporting inflammation.

## Introduction

Pancreatic cancer remains one of the most difficult cancers to treat [1]. The poor prognosis of patients with pancreatic cancer is largely due to early local invasion of adjacent structures and distant metastasis [2]. Most patients with pancreatic cancer are diagnosed with metastatic disease, and only 10% to 20% of patients with localized disease are resectable at the time of diagnosis [3]. Gemcitabine monotherapy or combination therapy with Erlotinib has become the standard chemotherapy to treat advanced pancreatic cancer; however, the median survival is 5–6 months [4]. Thus, it is important to find a therapeutic approach that aids in the prevention and/or treatment of pancreatic cancer metastasis.

Epithelial-to-mesenchymal transition (EMT), the process of cells losing their epithelial phenotype and acquiring a migratory mesenchymal phenotype, has been accepted to play an important role in cancer metastasis in several human malignancies including pancreatic cancer [5], [6]. EMT has been shown to negatively affect the overall survival of patients with pancreatic cancer [7]. Furthermore, EMT contributes to drug resistance in pancreatic cancer [8]. Thus, inhibition of EMT in pancreatic cancer cells may facilitate the development of an effective strategy in the prevention and/or treatment of metastatic pancreatic cancer.

Increasing evidence suggests that some Chinese herbal medicines, such as *Coptis chinensis* (Huang lian), *Scutellaria baicalensis* (Huang qin), *Camellia sinensis* (Lu cha), *Wedelia chinensis* (Peng qi ju) and Songyou Yin (a Chinese herbal compound), containing some bioactive compounds or a variety of phytochemicals, may reverse EMT and reduce metastasis [9]–[13]. Thus, these herbs can be utilized as complementary and alternative treatments and/or as adjuvant therapy for conventional cytotoxic therapies. QYHJ, a seven-herb Chinese medicinal formula and used for pancreatic cancer in China, has been shown to inhibit both tumor growth and metastasis in nude mice with pancreatic cancer cell xenografts [14]–[16]. In addition, QYHJ combined with conventional Western medicine has been shown to prolong survival time in patients with liver metastases from pancreatic cancer [17]. However, the underlying molecular mechanism remains unclear.

Here, we demonstrate that QYHJ inhibits both tumor growth and metastasis in nude mice with pancreatic cancer cell xenografts. The results indicated that QYHJ inhibited cancer-related inflammation in tumors by decreasing infiltration of tumor-associated macrophages and IL-6 production, which ultimately decreased EMT and cell invasion. Therefore, the present findings suggest that QYHJ is capable of inhibiting pancreatic cancer cell invasion and metastasis in human pancreatic cancer in part by reversing tumor-supporting inflammation.

## Materials and Methods

### Ethics Statement

All of the mouse experiments were conducted in accordance with the guidelines of the National Institutes of Health for the Care and Use of Laboratory Animals. The study protocol was also approved by the Committee on the Use of Live Animals in Teaching and Research, Fudan University, Shanghai. During establishment of a liver metastasis model and an orthotopic model of pancreatic cancer, the mice were anesthetized with 3% pentobarbital sodium (40 mg/kg) and all efforts were made to minimize suffering.

### Cell Lines and Mice

The human pancreatic cancer cell line BxPC3 was obtained from the American Type Culture Collection. SW1990HM, a highly metastatic human pancreatic carcinoma line, was subcloned from SW1990 by our group [18]. All of the cultured cells were grown in RPMI1640/10% fetal calf serum and maintained in a humidified 5% CO_2_ atmosphere at 37°C. All of the cell lines were regularly authenticated by checking their morphology and tested for the absence of mycoplasma contamination (MycoAlert, Lonza, Rockland, ME, USA).

Female BALB/c-nu/nu nude mice aged 4–6 weeks were obtained from the Shanghai Institute of Materia Medica, Chinese Academy of Sciences (Shanghai, China), and housed in laminar flow cabinets under specific pathogen-free conditions and provided with food and water *ad libitum*.

### Drugs and Reagents

QYHJ, a seven-herb Chinese medicinal formula, comprised *Scutellria barbata* (Ban zhi lian), *Heydyotis diffusa* (Bai hua she she cao), *Amorphophallus kiusianus* (She liu gu), *Coix lacryma-jobi* (Yi ren), *Gynostemma pentaphyllum* (Jiao gu lan), *Ganoderma luncidum* (Ling zhi) and *Amomum cardamomum* (Bai dou kou). QYHJ was prepared as previouly decribed [14]–[16]. Briefly, powders of QYHJ formula were produced by Jiang-yin Tianjiang Pharmaceutical Co, Ltd. To ensure standardization and maintain interbatch reliability of QYHJ, a high performance liquid chromatography (HPLC) chromatographic fingerprint was developed for its quality control. The fingerprint chromatograms of QYHJ formula was shown in Figure S1. The final decoction of QYHJ was prepared by dissolving the herbal powder in distilled water to the required concentration. The daily dosage for nude mice was 18 g/kg, as calculated according to the following human-mouse transfer formula Db = Da×(Rb/Ra)×(Wb/Wa)2/3, where D, R, and W represent dosage, shape coefficient, and body weight, respectively, and a and b represent human and mouse, respectively. Human recombinant interleukin-6 (IL-6) was obtained from R&D Systems (R&D Systems) and dissolved in sterile PBS containing 2% bovine serum albumin (BSA). The following antibodies were used: rabbit anti-E-cadherin, anti-vimentin, anti-N-cadherin and anti-Slug (Cell Signaling Technology, Beverly, MA); rabbit anti-CD68 (Santa Cruz Biotechnology, Santa Cruz, CA); goat anti-IL-6 (Santa Cruz Biotechnology, Santa Cruz, CA, USA); GAPDH (Epitomics, Burlingame, CA).

### Establishment of a Liver Metastasis Model in Nude Mice

A model of pancreatic cancer liver metastasis was established as described previously [18]. Briefly, nude mice were anesthetized with chloral hydrate; the spleen was exposed; and pancreatic cancer cells SW1990HM (5×10^6^/0.2 mL PBS) were injected into the spleen using a syringe. The spleen was returned to the abdomen, and the wound was closed in one layer with wound clips. Six weeks post-injection, the mice were examined grossly at necropsy for liver metastases. Next, we evaluated tumor metastasis by counting the number of metastatic colonies in one histological section of the mid-portion of each liver sample from each mouse and by determining the ratio of the metastatic area to the total area in histological sections from the midportion of each liver [19], [20].The metastatic to total area ratio was calculated using Adobe Photoshop version 7.0 (Adobe Systems) and ImageJ version 1.29 (National Institutes of Health) software, and the results are expressed as percentages.

### Orthotopic Model of Pancreatic Cancer

The orthotopic model of pancreatic cancer was established as previously described [21]. Briefly, 2×10^6^ SW1990HM pancreatic cancer cells per animal in 100 µL of phosphate-buffered saline were injected subcutaneously into the left flank of a nude mouse. Tumor sizes were measured 3 times weekly using a caliper. When tumors grown subcutaneously reached a size of 0.5 to 1 cm^3^, they were aseptically removed from the mice and transferred into Petri dishes containing 10 mL of phosphate-buffered saline. Macroscopically vital areas of the tumor were cut into 1-mm^3^ pieces using a scalpel. The mice were then anesthetized with 3% pentobarbital sodium (40 mg/kg) and all efforts were made to minimize suffering. A small transverse incision was created in the left abdominal flank, and the pancreas was carefully exposed. A pocket of 2 to 3 mm was prepared in the tail of the pancreas using the major spleen vein as a leading structure, and tumor pieces were inserted. The pancreas was then reinserted into the abdomen, and the abdominal wall was closed using 6-0 non-absorbable sutures.

### Enzyme-linked Immunosorbent Assay (ELISA)

Concentrations of serum IL-6 and TNF-α were measured using an ELISA. The blood sample was stored at room temperature for 30 min, centrifuged (12000 ×g) for 15 min, and then cryopreserved at −80°C. The concentrations of IL-6 and TNF-α were measured using a sandwich ELISA kit (DuoSet; R&D Systems, Minneapolis, MN, USA).

### Immunohistochemistry and Immunofluorescence

Immunohistochemistry (IHC) was performed as described previously [19]. Briefly, specimens of tumor tissue were fixed in 10% formalin and embedded in paraffin wax. Unstained 3-µm sections were then cut from the paraffin blocks for IHC analysis. The sections were stained with rabbit anti-vimentin (1∶200) and rabbit anti-E-cadherin (1∶100) and rabbit anti-CD68 (1∶200) at 4°C overnight. The secondary antibody and avidin-biotin peroxidase complex method was used according to the standard protocols provided by the manufacturer (Vector Laboratories, CA). An immunoglobulin-negative control was used to rule out non-specific binding. The procedures were performed by two independent investigators (PW and JZ) and one pathologist (JF), all of whom were blinded to the model/treatment type for the series of specimens. To quantitatively evaluate TAM infiltration in each group, we calculated the ratio of the area that was positive for CD68 staining to the total area of the histological sections from ten fields captured using light microscopy (200×). E-cadherin and vimentin expression were determined according to our previous report [16].

For immunofluorescence staining, cells and slides were fixed with 4% paraformaldehyde and 10% formalin, respectively. The slides were treated with 10 mM citrate buffer (pH 6.0) for antigen retrieval, according to the manufacturer's instructions. The cells or slides were then incubated overnight with anti-vimentin (1∶200), anti-E-cadherin (1∶100), anti-CD68 (1∶200) and anti-IL-6 (1∶200) at 4°C overnight. The cells or slides were then incubated with fluorescence-conjugated secondary antibody for 1 h. Finally, the cells were washed and mounted with mounting medium containing DAPI (Vector Laboratories). A negative control in which the primary antibody was omitted was used to test for antibody specificity. Images were captured using a confocal Leica fluorescence microscope.

### Western Blot Analysis

Western blot analysis was performed as described in our previous report [19]. Briefly, proteins were extracted from cultured cells and quantified with the bicinchoninic acid (BCA) assay kit (Pierce, Rockford, IL, USA) using BSA as a standard. Equal amounts of protein from different cells were separated using 10% SDS-PAGE and transferred to a nitrocellulose membrane (Bio-Rad). The membranes were blocked with 5% non-fat milk and incubated with primary antibodies. The target proteins were detected using an enhanced chemiluminescence (ECL) kit (Amersham Pharmacia Biotech, Uppsala, Sweden).

### Wound Closure Assays

Wound closure assays were performed as previously described [19]. Cells were plated in 6-well plates. The cell monolayer was wounded by manually drawing a furrow across the monolayer with a 1–10-µl pipette tip. The cell culture medium was then replaced with fresh medium, 100 ng/mL of IL-6 or vehicle was added as required, and wound closure was monitored at 48 h using phase contrast microscopy. After wounding, the wound area was quantified using Image J version 1.29 (National Institutes of Health) software. The experiments were performed in duplicate.

### Invasion Assay

Cell invasion was determined using Matrigel invasion chambers (Matrigel-coated membrane, BD Biosciences). Cells (1.0×10^4^) were seeded in serum-free medium in the upper chamber and allowed to invade toward the 10% FCS present in the lower chamber as a chemoattractant. After 48 h, cells that had traveled through and adhered to the underside of the membrane were counted as described previously [19].

### Statistical Analysis

Data are expressed as the mean ± standard deviation (SD). Statistical analyses were performed using analysis of variance (ANOVA) models and Student’s t-tests. A P-value <0.05 was considered statistically significant. All statistical analyses were performed using the SPSS 15.0 software package.

## Results

### QYHJ Suppresses Liver Metastasis from Human Pancreatic Cancer in vivo

**Figure 1:**
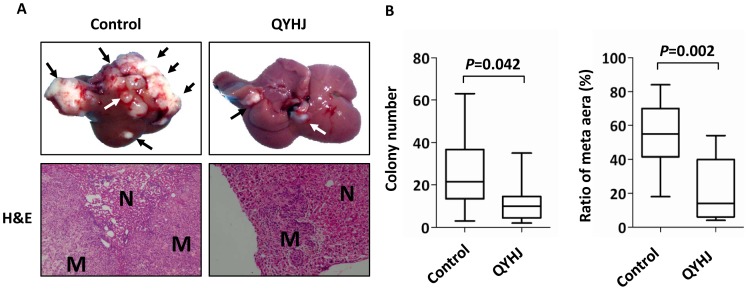
QYHJ inhibits liver metastasis from human pancreatic cancer in vivo. A. A liver metastasis model was established as described in Materials and Methods. Mice received a 28-day treatment of QYHJ or normal saline as a control. Mice were sacrificed 6 weeks post-injection, and their livers were harvested. Representative livers from nude mice are shown. H&E staining was performed on sections of metastatic tumors (M) and normal liver (N) tissues. The metastatic nodules are indicated by arrows. n = 6 per group. Original magnification, ×200. B. Liver metastases were quantified by counting the number of metastatic colonies in one histological section of the mid-portion of each liver sample from each mouse and by determining the ratio of the metastatic to the total area in histological sections from the mid-portion of each liver. The Student’s t-test was used to determine the statistical significance.

First, we confirmed the efficacy of QYHJ in suppressing liver metastasis of pancreatic cancer. Nude mice were inoculated intrasplenically with the human pancreatic cancer cell line SW1990HM and randomly divided into the QYHJ or the control group. One week after the randomization, mice in the QYHJ group were orally treated with QYHJ (0.2 mL, gavage, daily) while mice in the control group received normal saline as a control for 4 weeks (days 7–35). Six weeks post-injection, the mice were euthanized and their livers harvested. Both the QYHJ- and control-treated cells formed tumors when implanted in the spleen, but the mice treated with QYHJ developed significantly fewer liver metastases than did those treated with control normal saline. In addition, mice treated with QYHJ displayed fewer metastatic colonies and a decreased metastatic to total area ratio (Figures 1A and B). Together, these findings indicate that QYHJ could suppress liver metastasis in human pancreatic cancer.

### QYHJ Inhibits the Growth of Human Pancreatic Tumors in an Orthotopic Nude Mouse Model

**Figure 2:**
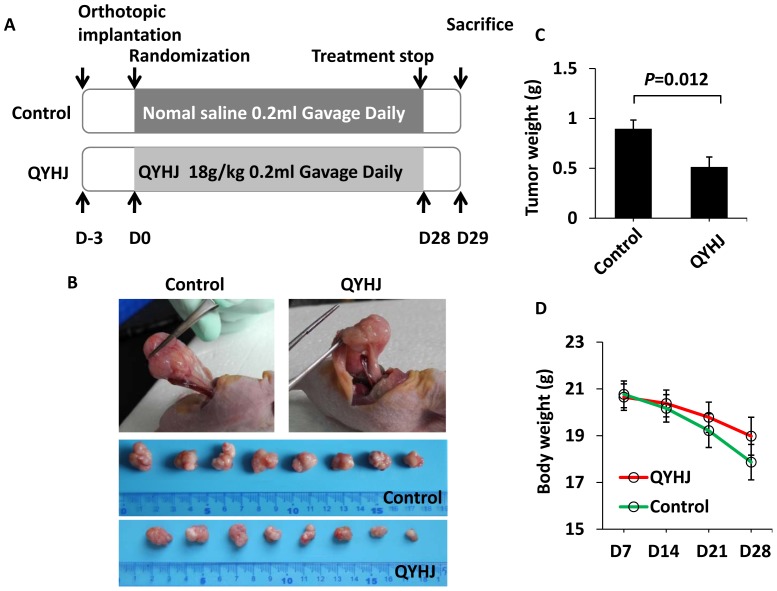
QYHJ inhibits the growth of human pancreatic tumors in an orthotopic nude mouse model. A. Flow chart of the experimental design and treatment schedule. B–D. Orthotopic models of pancreatic cancer were established as described in Materials and Methods. Three days post-establishment, mice were orally treated with or without QYHJ for 28 days, and then the tumors were removed and weighed. Photographs of orthotopic transplanted tumors from both groups are shown in B. The tumor weights are shown in C, and the body weight of the mice in both groups is shown in D. n = 10 per group. The Student’s t-test was used to determine the statistical significance.

We have shown that treatment with QYHJ reduces liver metastasis in nude mice inoculated intrasplenically with human pancreatic cancer cells. We extended this work to obtain additional insight regarding the effects of QYHJ on primary tumor in the pancreas and on liver metastasis. Nude mice received pancreatic implants with tumor fragments and then were divided randomly into two groups: one group received QYHJ and the other received normal saline as a control. The treatment was administrated for 28 days (Figure 2A). Tumors in the pancreas were identified in 8 of 10 mice (80%) in both groups. Liver metastases were not apparent in either group due to the cell type-specific “seed and soil” interactions governing the chronology and sites of metastatic targets and to the short observation period [22]. However, QYHJ significantly inhibited the growth of human pancreatic tumors in an orthotopic nude mouse model (Figure 2B). The average tumor weight was 0.90±0.25 g in the control group and 0.51±0.28 g in the QYHJ group (P = 0.036) (Figure 2C). In addition, QYHJ appeared to decelerate the loss of body weight in nude mice that were induced by tumor burden, although this difference was not significant (Figure 2D). These results suggested that QYHJ could inhibit pancreatic cancer in an orthotopic nude mouse model.

### QYHJ Inhibits EMT of Pancreatic Cancer Cells

**Figure 3:**
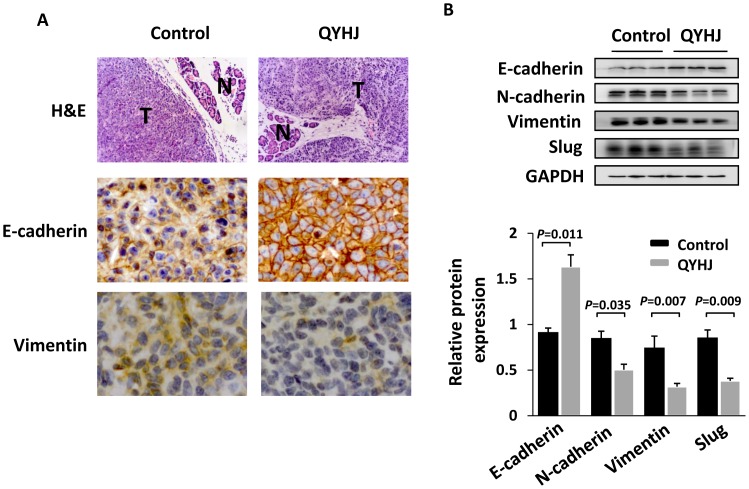
QYHJ inhibits EMT of pancreatic cancer cells. A. H&E staining and IHC staining with anti-Ecadherin and anti-vimentin antibodies were performed using sections of orthotopic transplanted tumors. N, normal pancreatic tissue; T, tumor. Original magnification, ×200. B. Western blot analysis was used to measure the expression of the EMT-related markers E-cadherin, N-cadherin, vimentin and Slug in cells derived from three representative xenograft samples from each group. The band intensities were measured by densitometry and the relative indicated protein expression was shown (lower).

EMT, a developmental program in which epithelial cells acquire a spindle cell morphology and exhibit cellular motility, has been proposed as a prerequisite for the invasion and dissemination of carcinoma cells and as a precedent to pancreatic cancer formation and metastasis [6], Therefore, we measured the expression of the major epithelial marker E-cadherin and the mesenchymal markers vimentin, N-cadherin and Slug in the tumor tissues of the two groups. Immunohistochemisty and/or western blot analysis revealed that the treatment of QYHJ induced the expression of E-cadherin and supressed the expression of vimentin, N-cadherin and the transcription factor Slug (Figure 3). These data suggested that QYHJ could inhibit EMT in human pancreatic cancer cells.

### QYHJ Suppresses Cancer-related Inflammation in Pancreatic Cancer

**Figure 4:**
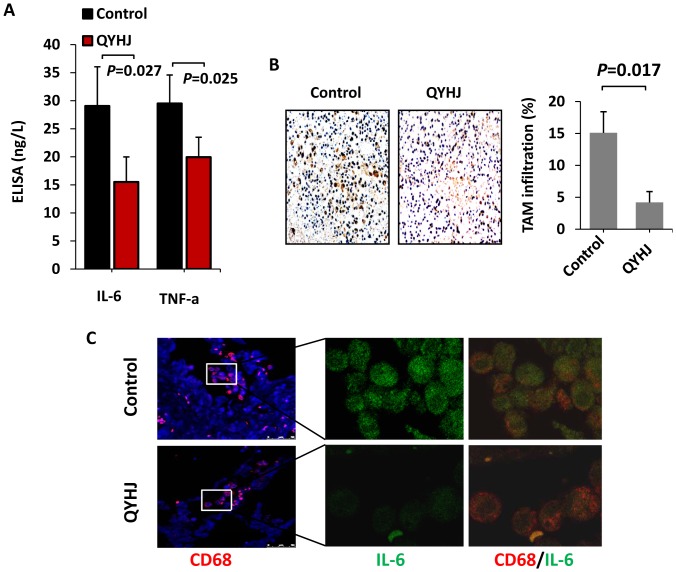
QYHJ suppresses cancer-related inflammation in pancreatic cancer. A. An orthotopic nude mouse model was established and treated with QYHJ or vehicle as described above. Blood was collected from each mouse, and the serum concentrations of the pro-inflammatory cytokines IL-6 and TNF-α were detected by ELISA. The Student’s t-test was used to determine the statistical significance. B. To evaluate TAM infiltration, CD68 staining was performed using IHC. Original magnification, 200×. TAM infiltration was evaluated quantitatively by calculating the ratio of the CD68-positive area to the total area in each field, and the mean value from ten fields under 200× microscopy was determined. *P<0.05. C. Confocal microscopy of pancreatic cancer tissues treated with anti-CD68 (red) and anti-IL-6 (green). Cell nuclei were counterstained with DAPI.

Numerous studies have indicated that cancer-related inflammation promotes the development of tumors [23], [24]. In addition, some pro-inflammatory cytokines, such as IL-8, IL-6 and TNF-a, have been shown to activate EMT and ultimately to facilitate cell invasion and metastasis [25]–[29]. Therefore, we used an enzyme-linked immunosorbent assay to examine serum levels of the two pro-inflammatory cytokines IL-6 and TNF-α in nude mice. Serum IL-6 levels were lower in mice treated with QYHJ (15.50 ng/L ±4.49 ng/L) compared with those receiving the control treatment (29.06 ng/L ±6.99 ng/L, P<0.01). In addition, serum TNF-α was diminished following treatment with QYHJ (19.94±3.55 ng/L vs 29.50±5.09 ng/L, P<0.01) (Figure 4A). These data demonstrated that QYHJ decreased the production of pro-inflammatory cytokines. Leukocyte infiltration is observed in most tumors and is involved in tumor invasion and metastasis [30]. Tumor-associated macrophages (TAMs) are an important component of the leukocyte infiltrate [31]. TAM infiltrates have been shown to correlate with tumor progression and a poor prognosis in cancer patients [32], [33]. We found that infiltration by TAM was blocked by QYHJ (Figure 4B). In addition, QYHJ decreased the expression of IL-6 in TAM (Figure 4C). Taken together, these results clearly showed that QYHJ reduced TAM infiltration and inhibited IL-6 expression, eventually suppressing cancer-related inflammation.

### IL-6 Induces Pancreatic Cancer Cell EMT

**Figure 5:**
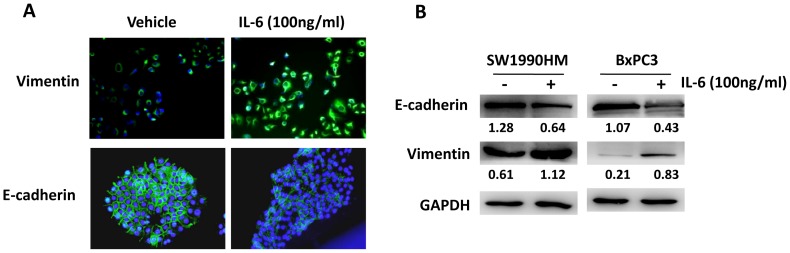
IL-6 induces EMT in pancreatic cancer cells. A. The pancreatic cancer cells SW1990HM and BxPC3 were treated with IL-6 (100 ng/mL) for 24 h. Cells were then stained to detect E-cadherin and vimentin (green). Cell nuclei were counterstained with DAPI. Original magnification, ×200. B. Pancreatic cancer cells were treated with IL-6 (100 ng/mL) for 24 h. Total protein was extracted, and the expression of E-cadherin and vimentin were detected by western blotting. GAPDH was used as a loading control.

To further confirm the correlation between EMT and the inflammatory tumor microenvironment, we evaluated the effects of IL-6 on pancreatic cancer cell EMT *in* *vitro*. SW1990HM, which exhibited a high level of vimentin expression, and BxPC3, which exhibited a high level of E-cadherin expression, were treated with recombinant human IL-6. IL-6 treatment resulted in increased vimentin expression in SW1990HM cells and decreased E-cadherin expression in BxPC3 cells, as detected by immunocytofluorescence (Figure 5A). These results were confirmed by western blot analysis (Figure 5B). Therefore, our results indicated that over-production of the pro-inflammatory cytokine IL-6 promoted pancreatic cancer cell EMT.

### IL-6 Promotes Pancreatic Cancer Cell Migration and Invasion

**Figure 6:**
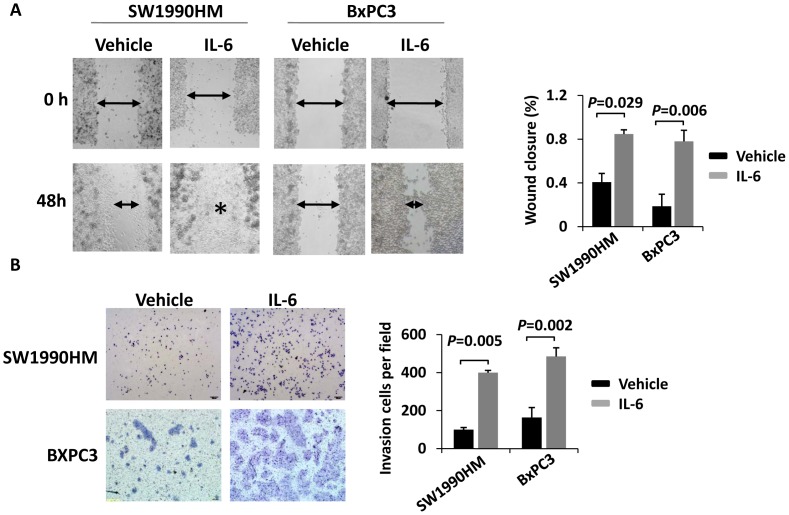
IL-6 promotes pancreatic cancer cell migration and invasion. A. Cell migration rates of pancreatic cancer cells treated with IL-6 (100 ng/mL) or vehicle only were compared via wound healing assays. Microscopic observations were recorded at 48 hours after scratching the surface of a confluent layer of cells. Asterisk indicates the wound was completely healed. B. The invasive capacity of pancreatic cancer cells treated with IL-6 (100 ng/mL) or vehicle only were compared using Matrigel invasion assays. The numbers of cells that traveled through the membrane were counted in 10 fields under a ×20 objective lens. Original magnification, ×200. The results represent the means ± SD of values obtained in three independent experiments. Statistical significance was calculated using the Student’s t-test.

**Figure 7:**
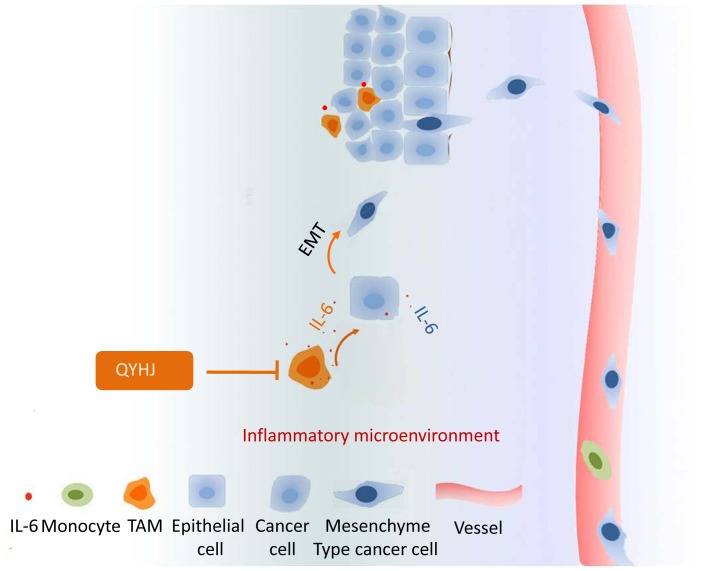
A schematic of the role of QYHJ in cancer-related inflammation and cell invasion. QYHJ targets cancer-related inflammation in pancreatic cancer via decreased TAM infiltration and IL-6 production. Because IL-6 can promote cancer cell migration and invasion by inducing EMT, the suppression of cancer-related inflammation by QYHJ can inhibit pancreatic cancer cell invasion and metastasis.

The effects of IL-6 on cell migration were further investigated using a wound-healing assay. The results showed that more cells moved into the scratch wound in IL-6 treated cells compared with cells that did not receive IL-6 treatment in both cell lines (Figure 6A). To examine the invasion capacity of IL-6-treated pancreatic cancer cells, we performed an *in vitro* invasion assay using a transwell chamber coated with matrigel. As expected, IL-6 treatment resulted in increased cell invasion *in vitro* in both cell lines (Figure 6B). These results combined with the observation that IL-6 induced pancreatic cancer cell EMT indicated that cancer-related inflammation was associated with an increased capacity for EMT and invasion (Figure 7).

## Discussion

The present results demonstrated that the Chinese herbal medicine QYHJ could inhibit pancreatic cancer cell invasion and metastasis via the suppression of cancer-related inflammation, especially the reduction of TAM-derived IL-6. These findings extend our previous observations regarding the importance of the tumor microenvironment as a key target of Chinese herbal medicine.

Traditional Chinese medicine (TCM), which is based on a unique theory and lone-term practical experience, is very popular in China. It has been reported that >90% of modern Chinese cancer patients have received some form of TCM during their treatment regimen [34]. The rates of TCM used by health care providers and the interest of patients outside of China continue to rise annually, especially within the field of oncology [35]. Application of TCM as an adjuvant cancer therapy has been reported to enhance the efficacy of both chemo- and radiotherapy and to help reduce the adverse effects of each treatment [36], [37]. Furthermore, the Chinese herbal medicines used in TCM have been recently recognized as an important source for novel drug development, including anti-cancer drugs [38], [39]. In the present study, we confirmed that QYHJ inhibited metastasis in pancreatic cancer; however, the underlying mechanisms remain unclear.

Recent studies have confirmed that cancer-related inflammation affects many aspects of malignancy, including the proliferation and survival of malignant cells, angiogenesis, metastasis, and response to treatment [24]. Tumor-promoting inflammatory cells include macrophage subtypes, mast cells, and neutrophils, as well as T and B lymphocytes. Macrophages constitute a major component of the immune cell infiltrate observed in virtually all malignancies [31]. It is accepted that, in general, cancer- and host cell-derived signals program macrophages to acquire an M2-like polarized and otherwise tumor-supportive phenotype [40]. In many cases, elevated numbers of TAMs are associated with a poorer prognosis [32], [33]. TAM can release growth factors (e.g., TGF-β), pro-inflammatory factors (e.g., IL-6) and enzymes (e.g., MMP2, MMP9) that promote angiogenesis, tumorigenesis, and metastasis [24]. All of these factors or enzymes can promote EMT. For example, the pro-inflammatory cytokines TNF-α, IL-6 and IL-8 can induce EMT and promote cancer metastasis [25]–[29]. In addition, a clinical investigation confirmed that these cytokines were elevated significantly in patients with pancreatic cancer and were associated with a poor prognosis [41]. Therefore, drugs that target cancer-related inflammation have the potential to re-educate a tumor-promoting inflammatory infiltrate or to prevent such cells from migrating to the tumor site. In the present study, we showed that QYHJ reduced TAM infiltration and effectively inhibited multiple pro-inflammatory cytokines such as TNF-α and IL-6, which suggested that QYHJ could inhibit cancer-related inflammation in pancreatic cancer.

During the process of EMT, extrinsic signals activate the intrinsic transcription factors Snail, Slug, Twist and ZEB1, which repress the expression of E-cadherin and promote the expression of vimentin and N-cadherin, such that cells change from a polarized epithelial phenotype to a migratory mesenchymal phenotype [42]. The “EMTed” carcinoma cells are thought to be responsible for seeding distant dissemination, eventually leading to cancer-related mortality [5], [7]. Therefore, we hypothesized that QYHJ inhibits the metastasis of pancreatic cancer cells, potentially by suppressing EMT. As we expected, our results confirmed that QYHJ treatment resulted in increased E-cadherin expression and decreased vimentin, N-cadherin and Slug expression, which indicated that QYHJ inhibited EMT of cells and suppressed cell invasion.

IL-6, which is produced mainly by mononuclear cells, macrophages and a smaller percentage of fibroblasts, endothelial cells, T and B lymphocytes, chondrocytes and amnion cells [43], is a pleiotropic cytokine that causes carcinogenesis, promotes tumor growth and facilitates tumor cell metastasis [25]–[27]. Accumulating evidence suggests that IL-6 is frequently elevated and thus is associated with a poor prognosis in many types of cancer, including pancreatic cancer [44], [45]. Additionally, new therapies targeting IL-6 are currently undergoing clinical trials [46]. Because we demonstrated that QYHJ reduced TAM infiltration and effectively inhibited IL-6 production, we sought to confirm whether the reduced IL-6 production was related to the suppression of EMT and invasive capacity of cells. Based on *in vitro* function studies, we confirmed that IL-6 could induce pancreatic cancer cell EMT and promote cell invasion. Therefore, our findings suggested that inhibition of IL-6 production by QYHJ might result in reduced EMT and invasion in pancreatic cancer.

QYHJ is a seven-herb Chinese medicinal formula. Similar to many other Chinese medicinal formulars, it is believed that the multiple components of the formula can affect multiple targets and exert synergistic therapeutic efficacies. However, essential compounds have not been identified in most formulae including QYHJ. Although QYHJ had an effect on cancer-related inflammation, i.e., an inhibition of TAM infiltration and IL-6 production, the precise underlying mechanism remains unknown. Eventhough, our study highlights the function of QYHJ in providing protection against metastasis in pancreatic cancer by targeting cancer-related inflammation, which may have important implications in our understanding of the efficacy of traditional Chinese medicine and in the discovery of promising future therapeutic approaches in pancreatic cancer.

## Supporting Information

Figure S1 **The fingerprint chromatograms of QYHJ formula.** A. High-performance liquid chromatography (HPLC) analyses were performed on an Agilent 1200 HPLC system with a photodiode array detector (DAD). An Lichrospher C_18_ column (200×4.6 mm,i.d., 5 µm, Jiangsu Hanbon Science & Technology Co., Ltd, Jiangsu, China) was used. The mobile phase consisted of acetonitrile (A) and 0.1% acetic acid aqueous solution (B) using a linear gradient program of 10–20% (A) in 0–40 min, 20% (A) in 40–60 min, and 20–50% (A) in 60–90 min. The flow rate was 1 ml/min and the column temperature was maintained at 50°C. 10 µL of standard and sample solution was injected in each run. The UV detection wavelength was set at 335 nm. B. Scutellarin, one of the major components of *Scutellria barbata* (Ban zhi lian), served as the reference standard.
(TIF)
