# Supplementary figures and images for: Targeting Cancer-Related Inflammation: Chinese Herbal Medicine Inhibits Epithelial-to-Mesenchymal Transition in Pancreatic Cancer
Source: PLoS One. 2013 Jul 29;8(7):e70334. doi: 10.1371/journal.pone.0070334 (PMC3726638; doi:10.1371/journal.pone.0070334)

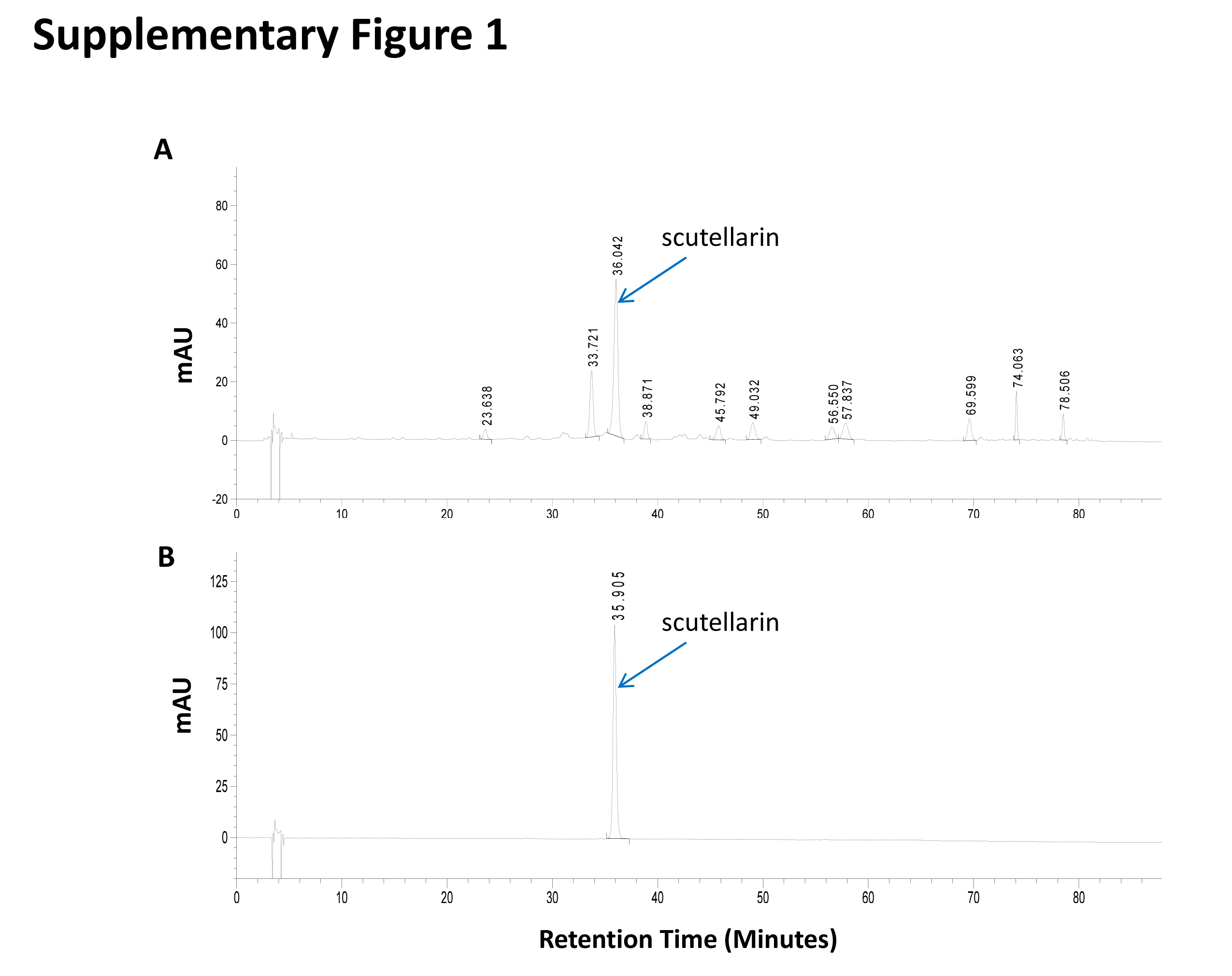

Supplement: Figure S1 — The fingerprint chromatograms of QYHJ formula. A. High-performance liquid chromatography (HPLC) analyses were performed on an Agilent 1200 HPLC system with a photodiode array detector (DAD). An Lichrospher C18 column (200×4.6 mm,i.d., 5 µm, Jiangsu Hanbon Science & Technology Co., Ltd, Jiangsu, China) was used. The mobile phase consisted of acetonitrile (A) and 0.1% acetic acid aqueous solution (B) using a linear gradient program of 10–20% (A) in 0–40 min, 20% (A) in 40–60 min, and 20–50% (A) in 60–90 min. The flow rate was 1 ml/min and the column temperature was maintained at 50°C. 10 µL of standard and sample solution was injected in each run. The UV detection wavelength was set at 335 nm. B. Scutellarin, one of the major components of Scutellria barbata (Ban zhi lian), served as the reference standard. (TIF) [file pone.0070334.s001.tif]
